# Multiorgan Involvement of Dormant Uveal Melanoma Micrometastases in Postmortem Tissue From Patients Without Coexisting Macrometastases

**DOI:** 10.1093/ajcp/aqad029

**Published:** 2023-04-13

**Authors:** Viktor T Gill, Emelie Norrman, Shiva Sabazade, Ali Karim, Emma Lardner, Gustav Stålhammar

**Affiliations:** Department of Clinical Neuroscience, Division of Eye and Vision, Karolinska Institutet, Stockholm, Sweden; Department of Clinical Pathology, Västmanland Hospital Västerås, Västerås, Sweden; Department of Clinical Pathology, Västmanland Hospital Västerås, Västerås, Sweden; Department of Clinical Neuroscience, Division of Eye and Vision, Karolinska Institutet, Stockholm, Sweden; St Erik Eye Hospital, Stockholm, Sweden; Department of Clinical Pathology, Västmanland Hospital Västerås, Västerås, Sweden; St Erik Eye Hospital, Stockholm, Sweden; Department of Clinical Neuroscience, Division of Eye and Vision, Karolinska Institutet, Stockholm, Sweden; St Erik Eye Hospital, Stockholm, Sweden

**Keywords:** Uveal melanoma, Postmortem, Ophthalmology, Pathology, Autopsy, Metastasis, Micrometastases, Macrometastases, Dormancy, Immunomagnetic separation

## Abstract

**Objectives:**

Almost half of all patients diagnosed with uveal melanoma will die of metastatic disease. This has been attributed to early seeding of micrometastases. We investigate the presence, density, organ involvement, and characteristics of micrometastases of uveal melanoma in tissue obtained at autopsy of patients with and without coexisting macrometastases.

**Methods:**

Patients diagnosed with primary uveal melanoma at a national referral center between 1960 and 2020 (n = 4,282) were cross-referenced with autopsy registers at nearby hospitals. Eleven patients were included. Formalin-fixed, paraffin-embedded tissue samples obtained during autopsy were examined with routine histology, immunohistochemistry, and immunomagnetic separation.

**Results:**

Micrometastases were detected in 5 of 5 patients with and in 5 of 6 patients without coexisting macrometastases. Micrometastases were identified in several sites, including lungs, kidneys, myocardium, and bone marrow. Their highest density per mm^2^ of tissue was seen in the liver. Of 11 examined patients, 2 had at least 1 BAP-1–positive metastasis. All micrometastases had immune cell infiltrates and no or very low proliferative activity.

**Conclusions:**

We demonstrate multiorgan involvement of apparently dormant micrometastases in patients with uveal melanoma. This suggests that micrometastases are present in nearly all patients diagnosed with primary uveal melanoma, regardless of coexisting macrometastases.

**KEY POINTS**

- A large proportion of patients with uveal melanoma die of metastatic disease even if the eye with the primary tumor is removed at an early stage.
- In this study, apparently dormant micrometastases of uveal melanoma were identified in 5 of 5 patients with and in 5 of 6 patients without concurrent macrometastases.
- This demonstrates that uveal melanoma macrometastases develop from micrometastases, which are present in nearly all patients with uveal melanoma.

## INTRODUCTION

Two percent of patients with uveal melanoma (UM) have radiologically detectable macrometastases at the time of primary tumor diagnosis.^1^ However, even with successful local outcome after treatment of the tumor eye, one-third of patients die of metastatic disease within 10 years of diagnosis.^2^ This is thought to be a result of early seeding of malignant cells from the primary tumor to distant organs, primarily the liver, where they can form subclinical micrometastases that remain dormant for many years before transitioning to proliferation and growth.^3,4^ In a previous study of 10 patients who died of metastatic UM, all had micrometastases in their liver.^5^ Furthermore, micrometastases have been detected in bone marrow (BM) aspirates from almost 40% of patients diagnosed with primary UM, and patients without BM micrometastases had worse—not better—overall and disease-specific survival.^6^ However, the presence of said micrometastases has not been proven outside of the BM in patients without coexisting macrometastases.

If the assumptions of early and frequent seeding of UM micrometastases are correct, and a period of dormancy precedes the onset of macrometastases, a large proportion of patients without macrometastases should have micrometastases, primarily in their liver.

We set out to test this hypothesis by collecting formalin-fixed, paraffin-embedded (FFPE) tissue from the liver and various other organs obtained at autopsy of patients with UM with and without coexisting macrometastases. The tissue was stained with immunohistochemical melanocyte and immune cell markers, digitally scanned, and also examined with immunomagnetic separation. Micrometastases were identified in 5 of 5 patients with and in 5 of 6 patients without coexisting macrometastases. This suggests that micrometastases are present in nearly all patients diagnosed with primary UM, regardless of coexisting macrometastases.

## MATERIALS AND METHODS

### Study Design and Patient Selection

The study was approved by the Swedish Ethical Review Authority (reference 2020–02172) and adhered to the tenets of the Declaration of Helsinki. Data from 4,282 patients diagnosed with primary UM at St Erik Eye Hospital, Stockholm, Sweden, between 1960 and 2020 were cross-referenced against archives of FFPE tissue at the St Erik Ophthalmic Pathology Laboratory, Stockholm, Sweden; the Karolinska University Hospital, Stockholm, Sweden; and the Västmanland County Hospital, Västerås, Sweden. As a result of a consensus between pathologists and clinicians about the general importance of autopsies, autopsy rates were high in the Swedish population in the decades preceding the turn of the millennium. In the 1970s and 1980s, more than 80% of all deceased Swedish inhabitants underwent autopsy. In the 1990s, this was reduced to 34%, followed by even lower rates in recent years.^7^ All available tissue samples from autopsies were collected and examined. During autopsy, pathologists had typically sampled tissue from a range of organs for histologic examination even if these had a gross normal appearance, including the brain, heart, lungs, liver, spleen, kidneys, BM (from the lower thoracic spine), and thyroid. From each organ, 1 or several pieces of tissue typically measuring 1 to several cubic centimeters are collected. If any pathology is suspected, including gross visible metastases, additional tissue will be sampled from that location. The registered cause of death was coded as either “possibly metastatic uveal melanoma” or “other than metastatic uveal melanoma.” The exact phrases used in the autopsy reports were not described herein, as they could lead to identification of individuals when combined with clinical data on patient sex and age.

Formalin-fixed, paraffin-embedded tissue was sectioned into 4-µm-thick sections, mounted on glass slides, and stained with H&E; CD markers CD3 (Dako Omnis, prediluted), CD20 (Dako Omnis, prediluted), and CD56 (Dako Link, prediluted); BRCA-associated protein 1 (BAP-1) (Santa Cruz Biotechnology); MIB1-IgG1 antibody against Ki67 (MIB1) (Dako Omnis, prediluted); melanoma antigen/melanoma antigen recognized by T cells 1 (MelA/MART1); and Sry-related HMg-Box gene 10 (SOX10).

For a patient to be classified as having macrometastases, any number of metastases had to be (1) clinically apparent before death (eg, palpable, leading to weight loss or jaundice or to abnormal liver enzyme levels in blood tests), (2) related to the cause of death, (3) visible with radiologic examinations or during gross autopsy, (4) more than 5 mm in diameter, or (5) within 3 mm from a macrometastasis during histologic examinations of tissue collected during autopsy.

Conversely, micrometastases were defined as being (1) clinically inapparent (not leading to symptoms or abnormal blood tests), (2) not related to the cause of death, (3) not detected by radiologic examinations or during gross autopsy, (4) 5 mm or less in diameter, and (5) separated from any macrometastasis by 3 mm or more of normal tissue.

All slides were examined by 2 pathologists (G.S. and V.T.G.). Growth patterns and sinusoidal involvement of liver metastases were evaluated according to international consensus criteria and previously described methods.^8,9^

In order to calculate the density of micrometastases, stained FFPE tissue sections were digitally scanned to NDPI file format at ×400 using a Nano Zoomer S360 (Hamamatsu Photonics). The area of each piece of tissue was measured with NDP view software (v2.9.25), allowing for calculation of the number of micrometastases per square millimeter.

Tissue from all patients without identifiable micrometastases in histologic and immunohistochemical (IHC) examinations was included for immunomagnetic separation (IMS). An equal number of patients with detected macrometastases as well as 1 patient with only micrometastases were included as positive controls. Micrometastases detected with IMS were not used for density calculations, as the method removes micrometastases from the surrounding tissue, precluding estimation of their distribution per square millimeter. We used the Mojosort Isolation Kit (BioLegend), Streptavidin Nanobeads (BioLegend), and a biotin-conjugated, SOX10 polyclonal, IgG rabbit antibody (Bioss Antibodies). A modified version of the Mojosort Isolation Kits Protocol 1 (Supplementary Figure 1; all supplemental materials can be found at *American Journal of Clinical Pathology* online) was used, together with the ACCUMAX Cell Detachment Solution (Capricorn Scientific). Deparaffinized tissue from each organ of interest was heated to 72 °C and treated with the EZ Prep Concentrate (Roche Diagnostics) and ULTRA Cell Conditioning Solution (Roche Diagnostics) at 95 °C for 52 minutes. The tissue was then immersed into buffer fluid in 1.5-mL and 5-mL polypropylene reaction tubes (Greiner Bio-One). A 70-µm cell strainer (Easystrainer Greiner Bio-One) was used for the filtration of the sample. After all steps of the IMS were performed, an additional centrifugation of the sample at 300*g* for 5 minutes was done in order to further increase the tumor cell yield in the pellet. A smear sample was thereafter made on Superfrost Plus Adhesion Microscope Slides (Epredia Netherlands). Each slide was then stained with H&E and MelA/MART1 clone A103. The cytologic features of isolated cells were evaluated by 2 pathologists (G.S. and V.T.G.), and consensus was needed before the cells were counted as metastatic tumor cells. The combination of SOX10 and MelA/MART1 was chosen since they complement each other in their binding sites, with SOX10 staining the nucleus and MelA/MART1 staining the cytoplasm of melanocytes.

### Statistical Analysis

*P* values below .05 were considered statistically significant, with all *P* values being 2-sided. For tests of continuous variables that did not deviate significantly from normal distribution (Shapiro-Wilk test *P* > .05), Student *t* tests were used. For nonparametrical data, Mann-Whitney *U* tests were used. All statistical analyses were performed using IBM SPSS statistics version 27 (SPSS) and GraphPad Prism version 9.3.0 (GraphPad Software).

## RESULTS

### Descriptive Statistics

Of 4,282 cross-referenced patients with primary UM, 12 had FFPE tissue samples obtained during autopsy available in the archives. One patient coded as having UM macrometastases was excluded because it was suspected that the metastases, having the appearance of adenocarcinoma with a lepidic growth pattern and immunohistochemical positivity for cytokeratin (CK) markers but negativity for CK20, MelA/MART1, and SOX10, more likely represented a metastatic lung cancer.

Of the 11 included patients, 5 (45%) were female and 6 (55%) were male. The mean (SD) age at the time of primary tumor diagnosis and death was 60 (16) years and 71 (12) years, respectively. The mean (SD) primary tumor diameter and apical thickness were 10.8 (3.7) mm and 5.1 (2.6) mm, respectively. Five of 9 (56%) available primary tumors had retained nuclear BAP-1 expression (nBAP-1 positive), whereas 4 (44%) had loss of expression (nBAP-1 negative). Ten patients had undergone enucleation for a choroidal melanoma, and 1 patient had a small iris melanoma that had been treated with plaque brachytherapy **TABLE 1**. All primary tumor treatments occurred within 1 to 3 weeks from diagnosis. No other non–basal cell malignancies were identified in the included 11 patients.

**TABLE 1:** Characteristics of Patients and Tumors

Patient No.	Sex	COD	Age at Diagnosis, y	Age at Death, y	PTT/PTD, mm	CBI	nBAP-1	Known Metastatic Disease Before Autopsy	Tissues Sampled During Autopsy	IMS
1	M	Possibly metastatic uveal melanoma	72	74	9.0/13.0	NA	NA	Yes	Liver	Yes
2	M	Other than metastatic uveal melanoma^a^	53	80	NA/NA	NA	NA	No	Liver Spleen	No
3	M	Possibly metastatic uveal melanoma	48	54	2.3/10.0	No	Pos	Yes	Bone marrow Heart Kidney Liver Lung Lymph node Spleen	No
4	F	Other than metastatic uveal melanoma^a^	67	76	6.9/8.5	Yes	Neg	Yes	Bone marrow Heart Liver Lung Spleen	No
5	F	Possibly metastatic uveal melanoma	77	85	3.9/12.0	No	Neg	Yes	Bone marrow Brain Heart Lung	Yes
6	M	Possibly metastatic uveal melanoma	61	72	4.0/4.0	No	Pos	No	Bone marrow Lung	Yes
7	M	Other than metastatic uveal melanoma^a^	74	74	9.0/18.0	No	Pos	No	Heart Kidney Liver Lung Spleen	No
8	F	Possibly metastatic uveal melanoma	66	70	6.0/10.0	No	Neg	Yes	Adrenal gland Bone marrow Heart Kidney Liver Lung Lymph node Pancreas Thyroid	Yes
9	M	Other than metastatic uveal melanoma^a^	55	60	3.0/11.0	No	Neg	No	Adrenal gland Heart Kidney Lung Lymph node Pancreas Prostate Spleen	Yes
10	F	Other than metastatic uveal melanoma^a^	40	78	NA/NA	No	Pos	No	Aorta Liver	Yes
11	F	Other than metastatic uveal melanoma^a^	54	60	2.5/11.0	No	Pos	No	Heart Kidney Liver Lung Uterus	Yes

CBI, ciliary body involvement; COD, cause of death; IMS, immunomagnetic separation; nBAP-1, nuclear BAP-1 expression in primary tumor cells; NA, not available; Neg, negative (indicating *BAP1* mutation); Pos, positive (indicating *BAP1* wild type); PTD, primary tumor diameter; PTT, primary tumor thickness.

^a^Including death from cardiovascular and pulmonary diseases but not uveal melanoma or other cancers.

### Histologic and Immunohistochemical Findings

Micrometastases were detected in histologic examination and IHC in 8 (73%) of 11 patients. Out of these 8 patients, 5 had focal liver lesions that were too small for definite classification in radiologic examinations before death. As these lesions were radiologically detectable, albeit unclassifiable, and later confirmed to be melanoma metastases upon histologic and immunohistochemical examinations, they were classified as macrometastases. The remaining 6 patients did not have any metastases that fulfilled the criteria for macrometastases (clinically apparent, related to the cause of death, detectable with radiologic examinations or during gross autopsy, ≥5 mm in diameter, or within 3 mm from another metastasis).

**FIGURE 1:**
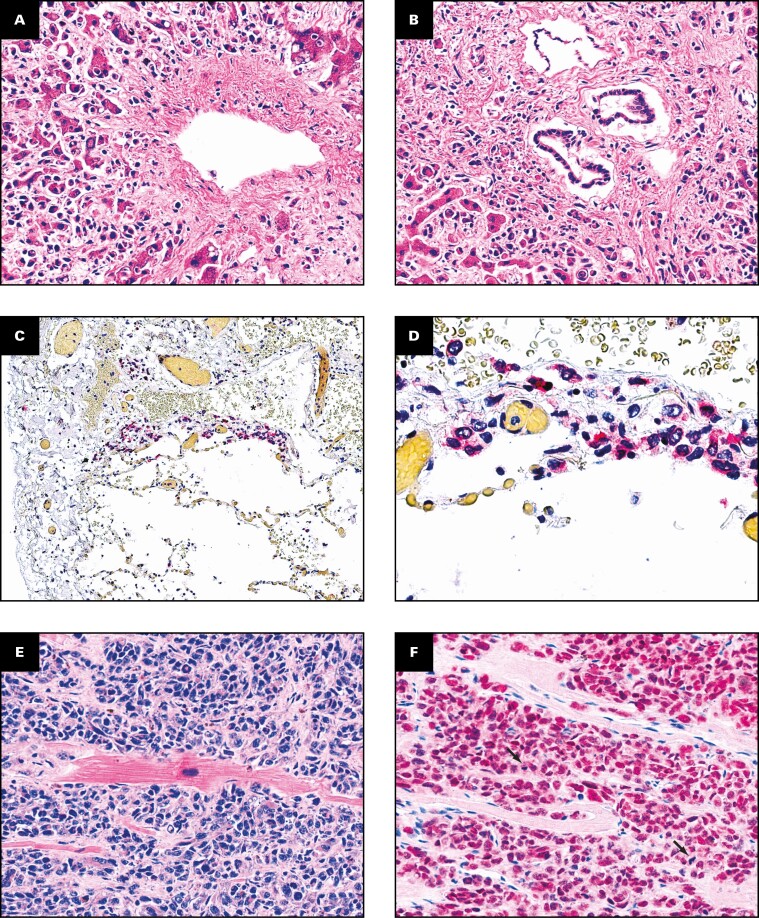

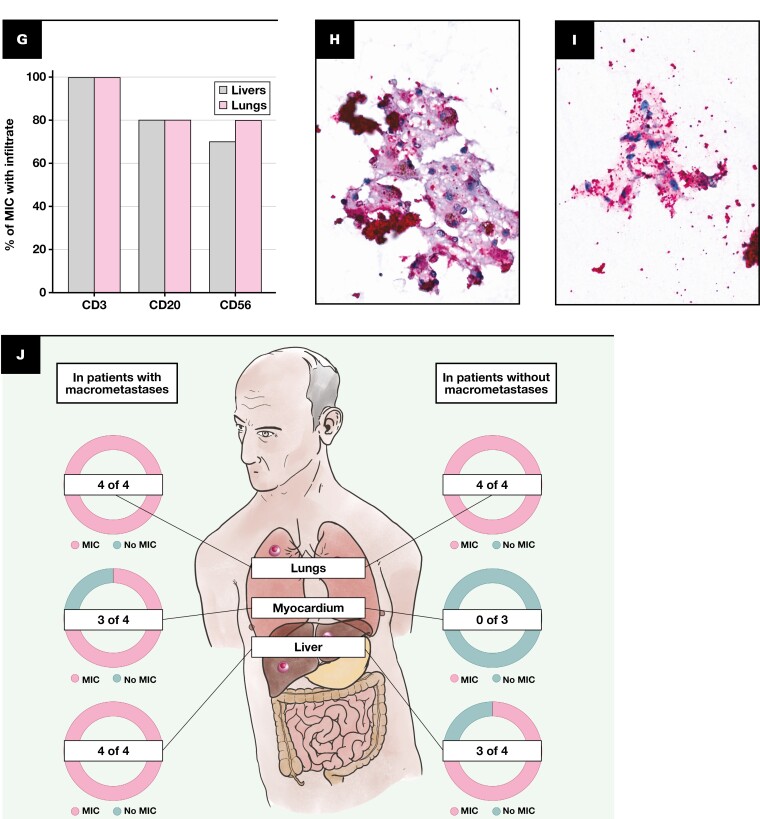
Micrometastases in the liver, lungs, and myocardium. **A**, Patient 1. Infiltration of hepatic sinusoids in an infiltrative/replacement growth pattern (H&E, ×20). **B**, Patient 1. Infiltration of hepatic sinusoids in a nodular fashion, resembling the desmoplastic and pushing growth patterns (H&E, ×20). Tumor cells in the lung of patient 3 with perivascular growth and growth in the interalveolar septa (asterisks), melanocyte marker MelA/MART1 (red chromogen; **C**, ×10; **D**, ×40). **E**, Patient 3. Tumor cells in the myocardium growing around cardiomyocytes (H&E, ×20). **F**, Patient 3. The same area of the myocardium stained with melanocyte marker SOX10. Scattered mitoses are present (arrows, ×20).

Micrometastases were of the epithelioid or spindle B-cell type and predominantly grew in an infiltrative manner, resembling the previously described replacement growth pattern, with a lesser degree of a nodular growth, resembling the previously described desmoplastic and pushing growth patterns **FIGURE 1A and FIGURE 1B**. Infiltration of the liver sinusoids was seen in both growth patterns. Scattered mitoses were mostly seen in the micrometastases bordering on macrometastatic size, with only a few mitotic figures being morphologically atypical. Pseudosinusoidal spaces were seen in all but 1 of the micrometastases in the liver. In lung parenchyma, micrometastases mainly displayed a perivascular growth pattern or grew in interalveolar septa **FIGURE 1C and FIGURE 1D**. The larger cell clusters tended to form nodular growth patterns. No mitotic figures or any reaction where fluid channels were formed, similar to the formation of pseudosinusoidal spaces in the liver, could be detected. Micrometastases in the heart mainly grew along cardiomyocytes and showed scarce mitotic figures **FIGURE 1E and FIGURE 1F**.

The median proportion of MIB1-positive tumor cells in micrometastases was 0% (range, 0%-3%); furthermore, no mitotic figures were seen in the smaller micrometastases. Of 11 examined patients, 2 (18%) had at least 1 BAP-1–positive metastasis. Tumor-infiltrating lymphocytes positive for CD3, CD20, and CD56 could be identified in most micrometastases in both lung and liver tissue. CD3-positive cells were seen in all examined micrometastases regardless of organ **FIGURE 1G**. When dividing the estimated volume of metastases with standard organ volumes from the literature, it was estimated that the average metastatic-to-normal tissue ratio was 3:1,000, corresponding to a total volume of about 30 mL in all examined organs combined (Supplementary Table 1).

### Presence and Characteristics of Micrometastases After Immunomagnetic Separation

Out of the included cases, 7 were selected for further examination with IMS. For patients without micrometastases in the previous step, liver tissue was used if available. Otherwise, BM, spleen, or lung tissue was used. In IMS, micrometastases were detected in 6 (86%) of the 7 patients, identifiable as clusters of atypical cells with irregularly shaped nuclei and positivity for MelA/MART1 **FIGURE 1H and FIGURE 1I**. The only patient in whom micrometastases could not be found was the 78-year-old woman who had been treated for iris melanoma (patient 10).

**FIGURE 2:**
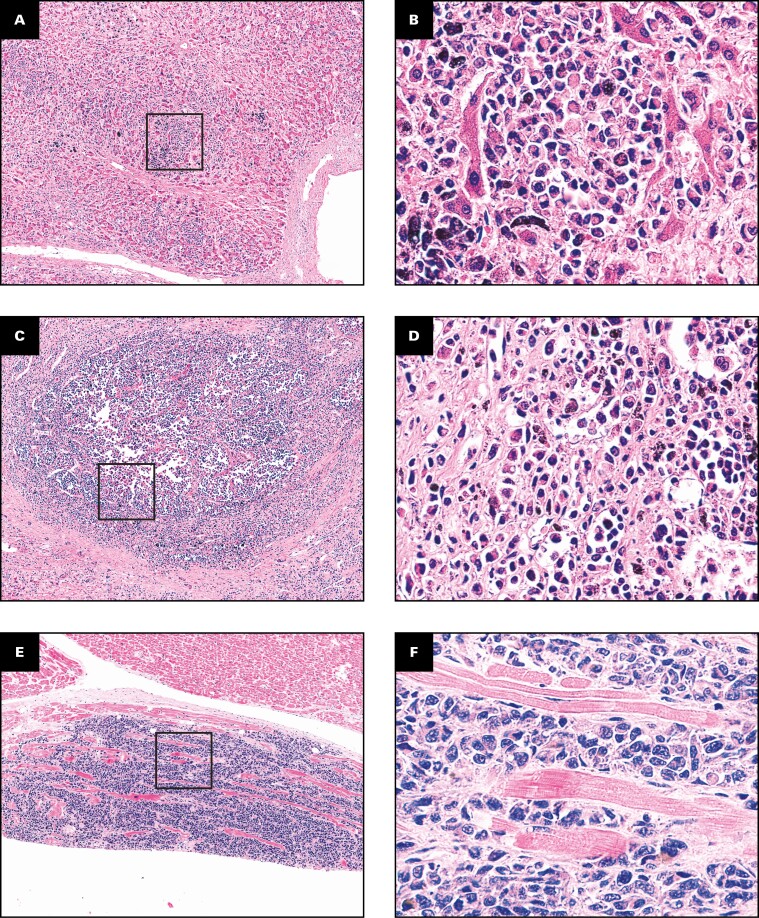

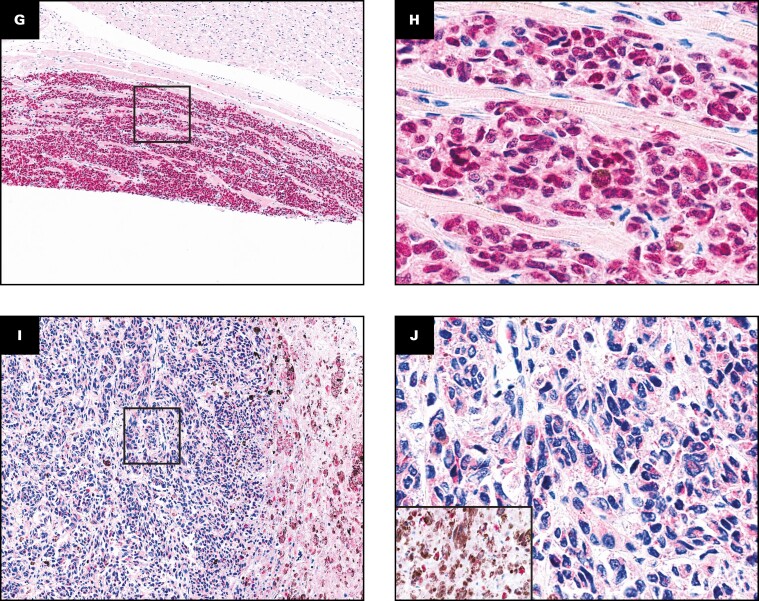
Examples of micrometastases of uveal melanoma, with framed area shown in paired image. **A**, **B**, Liver, patient 1. Micrometastases predominantly displayed an infiltrative or replacement growth pattern (H&E). **C**, **D**, Liver, patient 1. Nodular growth (previously described as desmoplastic and pushing growth pattern) was less common (H&E). **E**, **F**, Myocardium, patient 3. Sheets of infiltrative tumor cells (H&E). **G**, **H**, Myocardium, patient 3. The same area stained with melanocyte marker MelA/MART1. **I**, **J**, Liver, patient 3. All but 2 micrometastases were BAP-1 negative (inset, positive internal control). (**A**, **C**, **E**, **G**, **I**, ×5; **B**, **D**, **F**, **H**, **J**, ×40.)

In summary, micrometastases were detected in an additional 2 cases with IMS, for a total of 10 (91%) of 11 patients **FIGURE 1J** and **TABLE 2**. Further examples of the appearance of micrometastases are provided in **FIGURE 2** and in Supplementary Figures 2 to 4.

**TABLE 2:** Presence of Micrometastases in Each Examination Step

Patient No.	Suspected MAC in Clinical, Radiologic, or Gross Autopsy Examinations	MAC After IHC	MIC After IHC	MIC After IMS	Tissue Used for IMS
1	Yes	Yes	Yes	Yes	Liver
2	No	No	Yes	NA	NA
3	Yes	Yes	Yes	NA	NA
4	Yes	Yes	Yes	NA	NA
5	Yes	Yes	Yes	Yes	Bone marrow
6	No	No	Yes	Yes	Bone marrow
7	No	No	Yes	NA	NA
8	Yes	Yes	Yes	Yes	Bone marrow
9	No	No	No	Yes	Lung Lymph node Spleen
10	No	No	No	No	Liver
11	No	No	No	Yes	Liver Lung

IHC, immunohistochemistry; IMS, immunomagnetic separation; MAC, macrometastasis; MIC, micrometastasis; NA, not available.

### Density of Micrometastases

**FIGURE 3:**
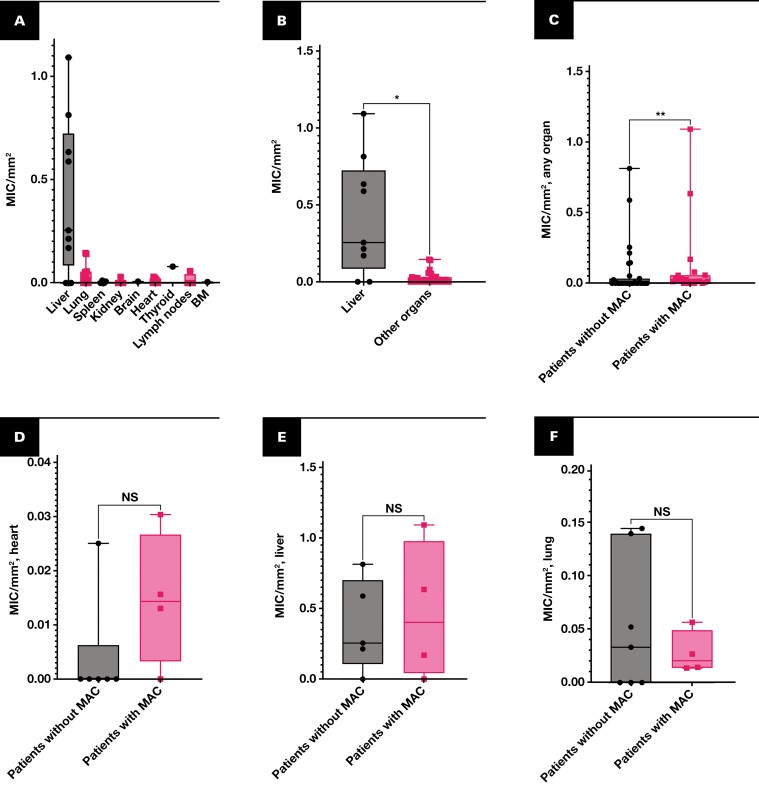
Boxplots, micrometastases (MIC) per square millimeter of examined tissue. **A**, **B**, The density of micrometastases was greater in the liver than in other organs (Mann-Whitney *U*, *P* = .002). **C**, Patients with concurrent macrometastases (MAC) had a higher density of micrometastases than patients without concurrent macrometastases (*P* = .002). **D–F**, There were no significant differences in the density of micrometastases in hearts (myocardium, *P* = .17), livers (*P* = .98), or lungs (*P* = .99) from patients with vs without concurrent macrometastases. Note that y-axis scale varies. BM, bone marrow; NS, nonsignificant. *P < .01. **P < .05.

The number of micrometastases per square millimeter of tissue was greater in the liver than in the other examined organs (Mann-Whitney *U*, *P* = .002) **FIGURE 3A and FIGURE 3B**. Patients with concurrent macrometastases had a higher density of micrometastases in their organs than patients without concurrent macrometastases (*P* = .002) **FIGURE 3C**. The density of micrometastases in myocardia (*P* = .17), livers (*P* = .98), and lungs (*P* = .99), however, did not differ between patients with and without concurrent macrometastases **FIGURE 3D, FIGURE 3E, and FIGURE 3F**.

Patients with primary tumor diameters greater than the median at the time of primary tumor treatment did not have a greater density of micrometastases in their livers (Mann-Whitney *U*, *P* = .99), lungs (*P* = .082), or myocardia (*P* = .063). Similarly, patients with primary tumors that were nBAP-1 negative did not have a greater density of micrometastases overall (*P* = .25) or in their livers (*P* = .20), lungs (*P* = .10), or myocardia (*P* = .21; Supplementary Figure 5).

## DISCUSSION

In this study, we demonstrate for the first time, to our knowledge, that micrometastases can be found in tissue outside of the BM obtained at autopsy from patients diagnosed with primary UM without detectable macrometastases. We also show that the liver is the predominant metastatic niche for micrometastases, followed by the lungs. All micrometastases were infiltrated by immune cells and had no or very low proliferative activity when examined morphologically or by an immunohistochemical proliferation marker, indicating possible dormancy. Interestingly, since not all of the included patients had previously detected metastases that would indicate an advanced late-stage disease, we were able to show an abundant intraluminal presence of metastatic tumor cells, arranged in clusters, in the liver sinusoids. These results seem to confirm the hypothesis of early seeding of micrometastases in UM. Furthermore, they are in line with the tumor cell dormancy concept and underline the scope for immune and adjuvant therapy of this aggressive cancer.^10-14^ From here on, the most important question should not be if micrometastases are present in patients with UM but rather by what mechanisms some micrometastases leave their dormancy and transition to lethal macrometastases.

Liver micrometastases were identified in a previous study of 10 patients with coexisting macrometastases.^5^ In that study, the metastases were divided into 3 categories based on size: stage I (≤50 µm), stage II (51-500 µm), and stage III (>500 µm, with no upper limit). We separated the metastases based on detectability by clinical and radiologic examinations, gross visibility during autopsy, and distance to the nearest other metastasis, with an upper threshold of a 5-mm diameter for micrometastases. This threshold was arbitrarily chosen and might be subject to debate. However, most of the micrometastases described herein were significantly smaller, which is underlined by the fact that in 2 patients we found tumor cells in parenchymatous organs with immunomagnetic separation that were not visible in any tissue when examined with light microscopy or IHC. Furthermore, we do consider the micro- and macrometastasis concept to have some benefit for the understanding of the metastatic process and dormancy and not least for future studies on this topic.

There are several limitations to this study. Foremost, the sample size was small, necessitating caution when interpreting the results. Second, we did not examine the molecular or cytogenetic features of primary tumors or metastases. Loss of BAP-1 expression correlates strongly to gene expression class, monosomy 3, tumor size, and ciliary body involvement. However, a more detailed investigation would have helped us clarify some of the genetic events involved in the metastatic progression.^4,15-17^ This should be pursued in future studies. Third, lung tissue samples are typically taken from peripheral parts of the lungs during autopsy, which correlates well with the fact that hematogenous metastatic spread to the lung most often manifests itself in a subpleural location.^18-20^ However, this means that we are unable, based on our material, to comment on whether or not micrometastases also occur in a more centralized position in the lung.

The main strength of this study is that we were able to prove the presence of micrometastases of UM in patients without macrometastases. Our access to material obtained over a long period of time made it possible for us to identify 6 patients whose cause of death was unrelated to metastatic UM, the otherwise main cause of death in this group of patients. Furthermore, since the study was performed on material collected during autopsy, we had access to relatively large volumes of tissue from a range of organs. The large amount of available tissue increased our chances to detect micrometastases and allowed us to assess their histologic growth pattern. We were also able to prove that immunomagnetic separation does indeed work for isolation of metastatic cells from aged FFPE tissue, as the oldest case subjected to this technique was autopsied in 1978.

## CONCLUSIONS

To our knowledge, this is the first study that demonstrates the presence of micrometastases outside the bone marrow in patients with UM without concurrent macrometastases. Micrometastases could be found in a range of organs, including lungs, kidneys, BM, and the myocardium, with their highest density being in the liver. All micrometastases had seemingly low proliferative activity and were infiltrated by immune cells. Our findings indicate that micrometastases can be found in nearly all patients diagnosed with primary UM, regardless of coexisting macrometastases. These results seem to confirm the hypothesis and mechanism of early seeding and dormancy of micrometastases in UM and would underline the scope for immune and adjuvant therapy.

## Supplementary Material

aqad029_suppl_Supplementary_Material

## Notes

Funding: This work was supported by the Swedish Cancer Society (grant 200798FK), the Swedish Eye Foundation (grant 2021-04-20), Karolinska Institutet (grant FS-2021-0010), and Region Stockholm (grant 20200356).
