# Supplementary material for: Multiorgan Involvement of Dormant Uveal Melanoma Micrometastases in Postmortem Tissue From Patients Without Coexisting Macrometastases
Source: Am J Clin Pathol. 2023 Apr 13;160(2):164–74. doi: 10.1093/ajcp/aqad029 (PMC10392366; doi:10.1093/ajcp/aqad029)
Supplement: aqad029_suppl_Supplementary_Material [file aqad029_suppl_supplementary_material.pdf]

Supplementary Material to

Multi-Organ Involvement of Dormant Uveal Melanoma Micrometastases in Postmortem Tissue From Patients Without Coexisting Macrometastases

Viktor T. Gill, MD[1,2], Emelie Norrman, BSc[2], Shiva Sabazade, MD[1,3], Ali Karim, MSc[2], Emma Lardner, BSc[3], Gustav Stålhammar, PhD[1,3]

[1]Department of Clinical Neuroscience, Division of Eye and Vision, Karolinska Institutet, Tomtebodavägen 18A, Stockholm, Sweden
[2]Department of Clinical Pathology, Västmanland Hospital Västerås, Sigtunagatan, 721 89 Västerås, Sweden.
[3]St. Erik Eye Hospital, Eugeniavägen 12, 171 64 Stockholm, Sweden.

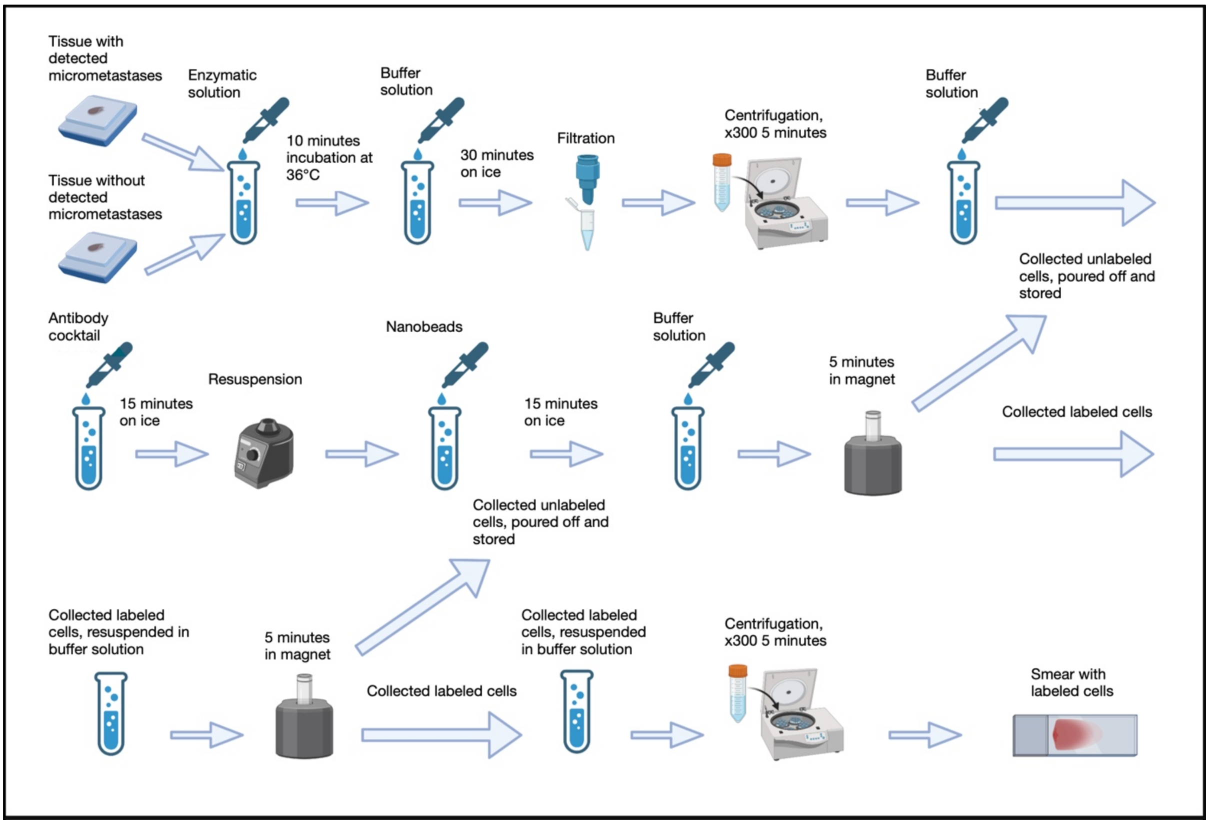

Supplementary figure 1. Overview of immunomagnetic separation work flow.

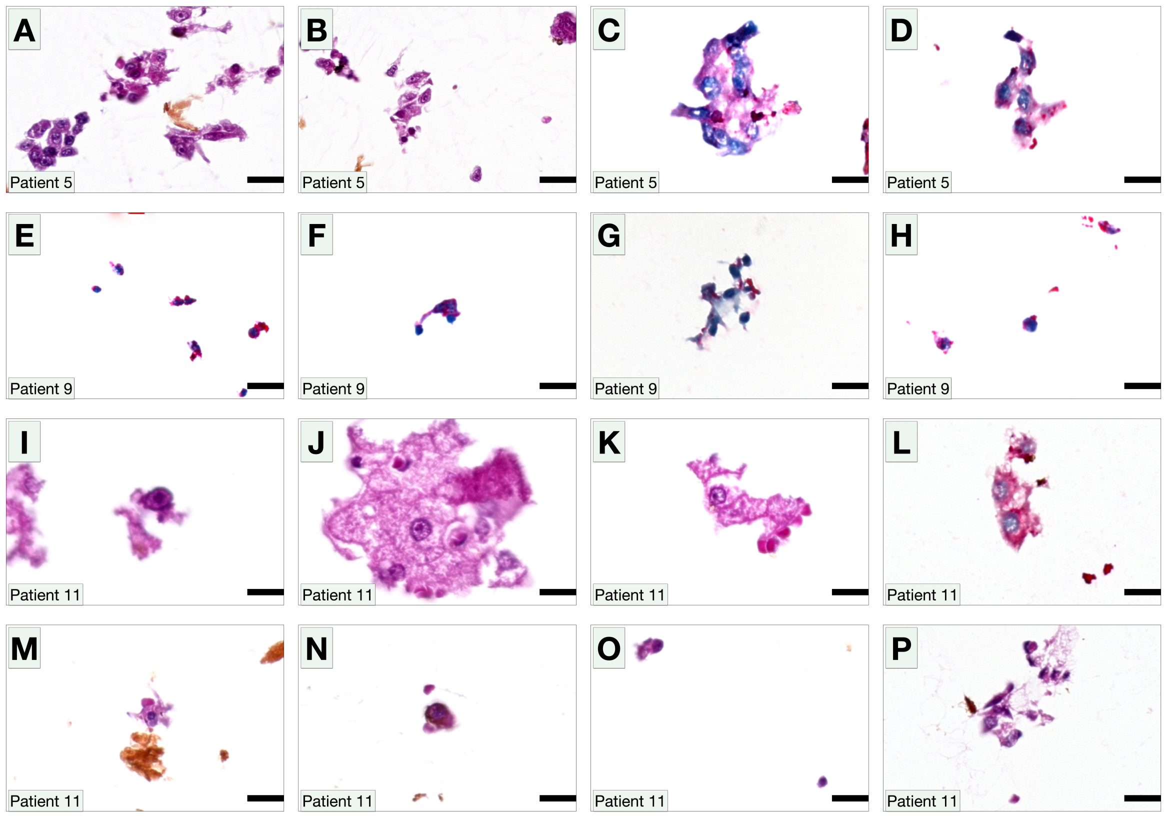

Supplementary figure 2. Metastatic tumor cells of uveal melanoma as seen after immunomagnetic separation. A-D bone marrow. E-H, M-P lung. I-L liver. Staining: A-B, I-K, M, N-P H&E. C-H, L MelA/MART1. Magnification: ×40. Scale bars: 10 µm.

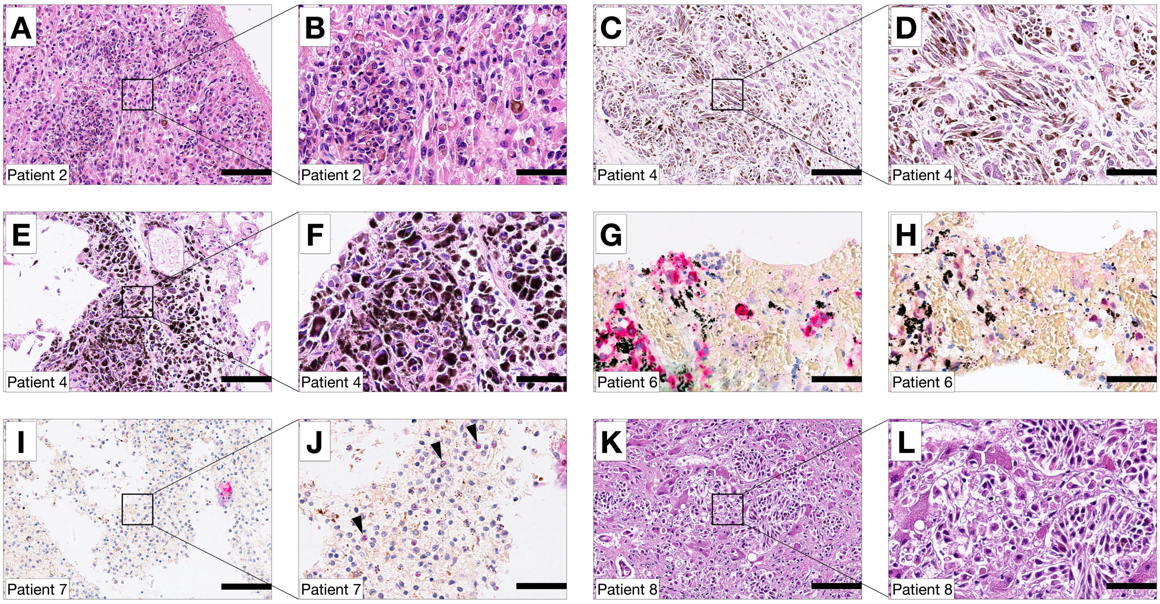

Supplementary figure 3. Examples of micrometastases of uveal melanoma. A-D, K-L liver. E-H lung. Magnification: A, C, E, G, H, I, K ×20. B, D, F, J, L ×40. Staining: A-F, K-L H&E. G, H MelA/Mart1. I, J SOX10. Arrowheads: Metastatic tumor cells. Scale bars: A, C, E, G, H, I, K 100 μm. B, D, F, J, L 50 μm.

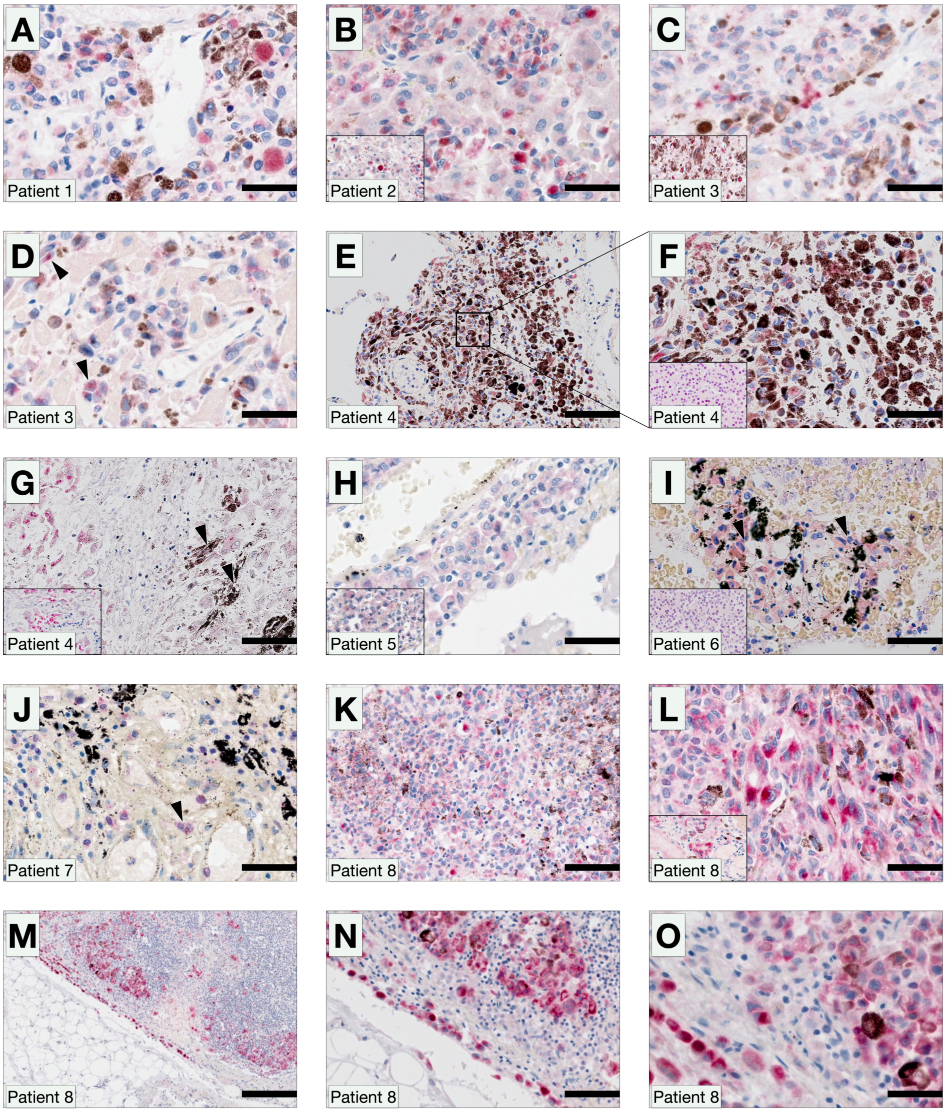

Supplementary figure 4. Of 11 examined patients, two (18%) had at least one BAP-1 positive metastasis. A-C, G liver. D, myocardium. E-K lung. L kidney. M-O, lymph node. BAP-1 positive cells: D, J. Inserts: B-C, G, H, L positive internal control. F, I positive external control. Magnification: A-D, F, H-J, L, O ×40. E, G, K, N ×20. M x5. Arrowheads: Metastatic tumor cells. Scale bars: A-D, F, H-J, L, O 50 µm. E, G, K, N 100 µm. M 285 µm.

Estimation of ocular versus systemic tumor burden

Considering the presence of micrometastases in various organs in all but one of the 11 patients, we estimated the aggregated volume of metastases in relation to primary tumors. The average metastatic-to-normal tissue ratio was 3:1000, corresponding to a total volume of metastatic tissue of 30 000 mm^3 (30 mL) in all examined organs, or about two tablespoons (supplementary table).

This was a significantly higher volume than the mean volume of primary tumors at 472 mm^3 (SD 480, Mann-Whitney *U* $P<0.001$). In linear regressions of primary tumor volumes and the volume of metastatic tissue in organs with at least 4 available measurements, the primary tumor volume did not correlate with the volume of metastatic tissue in livers ($R^2=0.20$, $P=0.55$), lungs ($R^2=0.11$, $P=0.67$), spleens ($R^2=0.01$, $P=0.86$), kidneys ($R^2=0.05$, $P=0.78$), bone marrow ($R^2=0.25$, $P=0.40$), with the average metastatic volume per organ ($R^2<0.01$, $P=0.90$), or with the sum of metastatic tissue volume ($R^2<0.01$, $P=0.91$).

Supplementary table. Average volumes of metastases in examined organs.

Organ	Average volume of metastases, mm^3 (SD)	Average organ volume, mm^3	Average proportion of organ volume affected
Adrenal gland	0.0 (0.0)	7200	0.0
Aorta (ascending)	0.0 (0.0)	3400	0.0
Bone marrow	0.2 (0.4)	1500000	<0.1
Brain	<0.1 (0.0)	1200000	<0.1
Kidney	<0.1 (<0.1)	260000	<0.1
Larynx	0.0 (0.0)	25000	0.0
Lgll (thoracic & abdominal)	4607.6 (6397.7)	34000	0.1
Liver	17857.3 (35578.5)	1500000	<0.1
Lung	5770.5 (19986)	6000000	<0.1
Myocardium	1331.1 (4414.6)	200000	<0.1
Pancreas	0.0 (0.0)	75000	0.0
Prostate	0.0 (0.0)	25000	0.0
Spleen	<0.1 (0.1)	200000	<0.1
Thyroid	<0.1 (0.0)	6000	<0.1
Uterus	0.0 (0.0)	250000	0.0
Sum	*29567 (66 377)*	*11285600*	*0.0026**

SD, standard deviation. *Sum of average volumes of metastases divided by sum of organ volumes.

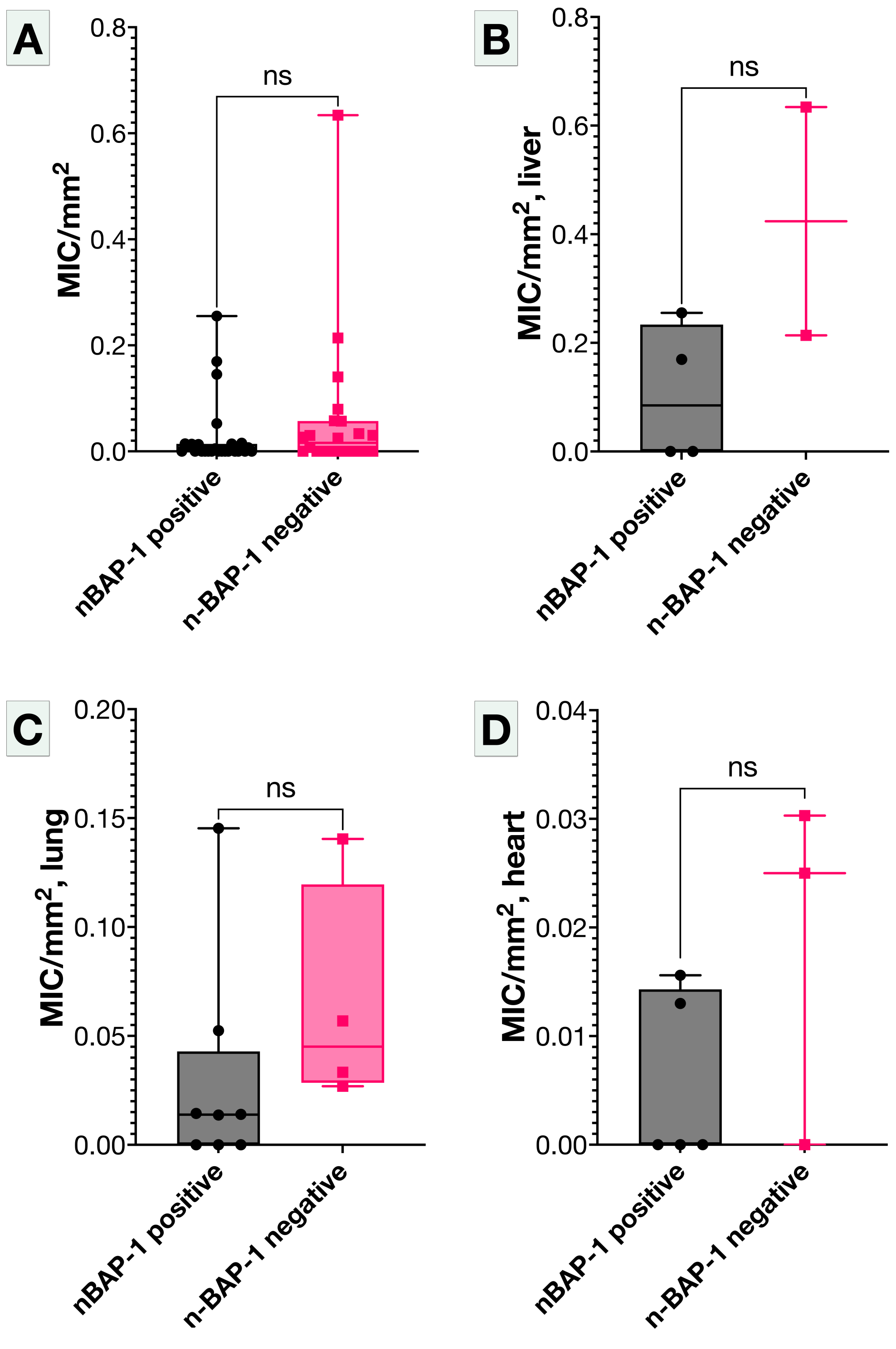

Supplementary figure 5. Density of micrometastases (MIC) in relation to primary tumor nuclear BAP-1 expression (nBAP-1). A) Patients with nBAP-1 negative primary tumors did not have greater density of MIC, all organs included (Mann-Whintey *U* P=0.25). Similarly, the density of MIC in B) livers (P=0.20), C) lungs (P=0.10), or D) myocardia (P=0.21) did not differ between patients with nBAP positive and negative primary tumors. ns, non-significant.
